# Prevalence, Etiology, Risk Factors, and Complications of Facial Nerve Palsy at King Abdulaziz Medical City: A Multicenter Study

**DOI:** 10.7759/cureus.53403

**Published:** 2024-02-01

**Authors:** Ali Ragaban, Lana Alsharif, Nada A Alshaikh, Rafaa J Jafar, Ziyad Hemeq, Muhammad A Khan, Raidaa A Gharawi, Taghreed Aldosary

**Affiliations:** 1 Faculty of Medicine, King Abdulaziz University, Jeddah, SAU; 2 Department of Medical Rehabilitation Sciences/Physiotherapy, Faculty of Applied Medical Sciences, Umm Al-Qura University, Makkah, SAU; 3 College of Medicine, Jazan University, Jazan, SAU; 4 Faculty of Dentistry, King Abdulaziz University, Jeddah, SAU; 5 Medical Education, King Saud Bin Abdulaziz University, Jeddah, SAU; 6 Dental Clinic, Jeddah University Medical Centre, Jeddah University, Jeddah, SAU; 7 Medical Sciences-Oral Biology, Ministry of the National Guard-Health Affairs, King Abdulaziz Medical City, Jeddah, SAU

**Keywords:** facial asymmetry, brow drooping, facial nerve (cn vii) paralysis, prevalance, bells palsy, facial nerve paralysis

## Abstract

Background: Facial nerve palsy is a condition of nerve damage that results in impaired facial movement on one or both sides of the face.

Objectives: This multicenter study aimed to determine the prevalence of facial nerve palsy and evaluate the association between its risk factors and complications to identify its etiology among patients admitted to King Abdulaziz Medical City in Jeddah and Riyadh, Saudi Arabia, between 2016 and 2023.

Methods: A retrospective cross-sectional study was conducted to obtain data from medical records using the best care system for patients with facial nerve palsy. Data were analyzed using IBM SPSS Statistics for Windows, Version 20.0 (Released 2011; IBM Corp., Armonk, New York, United States), Chi-square test, t-test, and ANOVA. The level of statistical significance was set at p<0.05.

Results: The study involved 123 patients, with 0.0164% prevalence. Bell's palsy was the most common etiology, accounting for 81.8% of cases, followed by head injuries, dental trauma, otitis media, stroke, and head and neck tumors. Obesity was the most significant risk factor, followed by upper respiratory problems. Hypertension and diabetes exert similar effects. Facial asymmetry, ophthalmic complications, and eye twitching were the most common complaints followed by speech difficulties, psychological and social effects, mouth twitching, and synkinesis.

Conclusion: Facial nerve palsy is common in this region. We recommend health education sessions to increase public awareness and provide preventive strategies to reduce the complications of facial nerve damage. We recommend further research on the association between the risk factors and complications of facial nerve palsy.

## Introduction

Facial nerve palsy (FNP) is a common condition that can have a significant impact on a patient’s quality of life [1]. FNP refers to impaired facial movements caused by nerve damage that can manifest on one or both sides of the face [2]. It affects about 15-40 per 100,000 adults annually [1]. Both children and adults develop peripheral FNP [3]. In children, FNP can be congenital or acquired [4]. FNP can cause a variety of symptoms, including drooping of the brow, incomplete eyelid closure, mouth drooping, difficulty closing the mouth, dry eyes, sound sensitivity, impaired taste, and ear pain [5].

According to a study in Saudi Arabia, facial palsy prevalence was 26.3% [6], which is significantly higher than the 0.7% reported in New York City [7]. Infections, inflammation, head injuries, tumors, surgeries, autoimmune diseases, chronic otitis media, local anesthesia, and strokes can cause FNP [2,8-11]. Bell's palsy is the most common diagnosis of FNP and acute mono-neuropathy [9]. Bell's palsy is responsible for 70% of FNP cases [12]. Trauma is the second most common cause accounting for 10-23% of cases [12]. FNP had increased during the coronavirus disease 2019 (COVID-19) pandemic, possibly linked to COVID-19 infections [13].

In addition to the aforementioned causes, various risk factors are linked to FNP, which include obesity, myasthenia gravis, hypertension, Lyme disease, multiple sclerosis, and diabetes [14]. People with respiratory issues, weakened immune systems, and pregnant women are more likely to develop facial palsy; pregnant women are at a higher risk with rates three times higher than the general population [15,16]. FNP can lead to complications such as ophthalmic issues (exposure keratitis, corneal dryness, ulceration risk), facial asymmetry, synkinesis, and hemifacial spasm [4].

In Saudi Arabia, there is a lack of knowledge regarding the prevalence of FNP and its associated risk factors and complications. This study aimed to determine the prevalence of FNP and investigate the link between risk factors and complications in Saudi Arabia. This will help in enhancing FNP knowledge and creating better management, diagnostic strategies, planning, and allocating resources for medical services and research, thereby improving patient outcomes.

## Materials and methods

This was a retrospective cross-sectional study approved by the Institutional Research Board of King Abdulaziz Medical City (approval number: IRB/1998/23). Data were obtained from the medical records department at the outpatient clinics of King Abdulaziz Medical City in Jeddah and Riyadh, Saudi Arabia, by using BESTCare EMR Solution (ezCaretech Co., Ltd, South Korea). The inclusion criteria were patients with facial palsy who attended King Abdulaziz Medical City between 2016 to 2023. All ages and genders were included. There were no exclusion criteria.

A data collection sheet (Appendix 1) was used to record the data, including predetermined variables such as demographics, symptoms, complications, risk factors, and etiologies. Complete information from the data sheet was entered into MS Excel (Microsoft Corporation, Redmond, Washington, United States) prior to the analysis.

Descriptive statistics were calculated using count/frequency and percentages for categorical variables such as sex, educational level, marital status, etiologies, risk factors, and complications. Mean and standard deviation were used to present the numerical variables (age and income). Regarding inferential statistics, when comparing two categorical variables, either Chi-square test or Fisher’s exact test was used, whichever was appropriate. A t-test and analysis of variance were used to compare numerical and categorical variables. Statistical significance was set at p<0.05. IBM SPSS Statistics for Windows, Version 20.0 (Released 2011; IBM Corp., Armonk, New York, United States) was used for data analysis.

## Results

The study included 123 patients with 66 (53.7%) females and 57 (46.3%) males. The male-to-female ratio was 19:22. Overall prevalence of FNP was 0.0164% as shown in Table 1. Patients' ages ranged from one year to 81 years, with a mean age of 40.1 ± 18.77. The largest age group was 20-29 years, representing 24.8% of the sample. The distribution was similar for the 30-39, 40-49, and 50-59 age groups, representing approximately 14.6-15.4% for each age group. The age groups of 60-69, 70-79, and 80-89 accounted for 15.2%, 6.7%, and 1% of the participants, respectively. The mean age of males was 40.9 years, while that of the women was 39 years. The relationship between sex and age was not statistically significant (p=0.660). The majority of patients were married (54.2%) followed by 31.7% of single patients (Table 1).

**Table 1:** Demographic characteristics of the patients

Demographic Characteristics		Frequency	Percentage
Gender (n=123)			
	Female	66	53.7
	Male	57	46.3
Marital status (n=120)			
	Divorced	1	0.8
	Married	65	54.2
	Single	50	41.7
	Widow	4	3.3

The most common etiology of FNP in this study was Bell's palsy, followed by head injury, dental trauma and parotidectomy, acute and chronic otitis media, stroke, head and neck tumors, and surgery trauma local anesthesia. Three percent of cases involved diverse and less common etiological factors and were included in the "others" category (data not presented in table). The most common presenting risk factor was obesity (39.2%) followed by upper respiratory problems (23.1%). Hypertension and diabetes exhibited similar prevalence rates, affecting 22.1% of participants (Table 2). Multiple sclerosis and Lyme disease were not observed in our study population. Some of the patients had multiple risk factors: 17.2% had both diabetes and hypertension, and 4.9% had diabetes, hypertension, and obesity. 

**Table 2:** Risk factors found among the patients COVID-19: coronavirus disease 2019

Risk Factors		Frequency (N=123)	Percentage
Obesity (n=120)			
	No	73	60.8
	Yes	47	39.2
Myasthenia gravis (n=122)			
	No	120	98.4
	Yes	2	1.6
Hypertension (n=122)			
	No	95	77.9
	Yes	27	22.1
Diabetes (n=122)			
	No	95	77.9
	Yes	27	22.1
Lyme disease (n=122)			
	No	122	100.0
	Yes	0	0.0
Multiple sclerosis (n=122)			
	No	122	100.0
	Yes	0	0.0
Upper respiratory problems (n=121)			
	No	93	76.9
	Yes	28	23.1
Immunocompromised (n=122)			
	No	105	86.1
	Yes	17	13.9
Pregnancy (n=122)			
	No	116	95.1
	Yes	6	4.9
COVID-19 (n=122)			
	No	118	96.7
	Yes	4	3.3
Cold season			
	No	102	82.9
	Yes	21	17.1
Hot season			
	No	48	39.0
	Yes	75	61.0

The most common presenting complaints were facial asymmetry, ophthalmic complications, and eye twitching. Five percent of patients had speech difficulties; psychological and social effects had the same prevalence as patients with mouth twitching. The least common complication was synkinesis (Table 3).

**Table 3:** Distribution of common complications among the patients

Complications		Frequency (N=123)	Percentage
Ophthalmic complications (n=121)			
	No	104	86.0
	Yes	17	14.0
Facial asymmetry (n=121)			
	No	2	1.7
	Yes	119	98.3
Synkinesis (n=120)			
	No	118	98.3
	Yes	2	1.7
Eye twitching (n=120)			
	No	115	95.8
	Yes	5	4.2
Mouth twitching (n=120)			
	No	117	97.5
	Yes	3	2.5
Speech difficulties (n=119)			
	No	113	95.0
	Yes	6	5.0
Social effects (n=119)			
	No	116	97.5
	Yes	3	2.5
Psychological effects (n=119)			
	No	116	97.5
	Yes	3	2.5

This study uncovered the association between the risk factors and complications of FNP. Eye twitching and hypertension had a statistically significant correlation (p=0.009), and speech difficulties were also associated with both hypertension (p=0.023) and diabetes (p=0.003) statistically significantly. Immunocompromised patients also exhibited a significant relationship with eye twitching (p=0.020).

## Discussion

This study included 123 participants with an overall prevalence of facial paralysis of 0.0164%. This was lower than the results of studies conducted in Arar, northern Saudi Arabia (26.3%) [6] and southern Nigeria (0.95%) [17]. In the current study, females were preponderant, which was similar to a previous study [6]. However, according to other studies, facial palsy affects both sexes equally [9,12]. The mean age of the examined patients was 40.1±18.77 years. In contrast, Wasan et al.'s study involved a younger generation with a mean age of 32.1 years [6]. In this study, individuals aged 20-29 years were the most affected. Similarly, Alanazi et al. observed that people between the ages of 20 and 30 years were the most affected [4]. In a study from Nigeria, individuals aged 50-59 were affected more [17].

There are several etiologies of FNP [9]. Of the cases of one-sided FNP, 70% are caused by Bell's palsy [13]. Bell's palsy emerged as the primary cause, accounting for a significant number of cases (81.8%) in this study. Bell's palsy was the cause of 81% of cases of FNP in a study from Romania [18].

Trauma, which accounts for 10-23% of cases, is the second cause of facial paralysis [13]. The current study also had similar results; Bell’s palsy was followed by trauma (head injuries, dental trauma) (4.1% and 3.3%, respectively). Acute and chronic otitis media was found in 2.5% of cases in our investigation, which is comparable to the 3.7% reported in an Arar study that reported acute otitis media [6]. Acute and chronic otitis media accounted for 13.1% of the infectious causes in a study conducted by Rodrigues et al. [19]. However, Lamina et al. found otitis media in 76 (12.8%) cases of facial palsy, which was consistent with North Western University Medical School's findings that otitis media rarely causes facial palsy [20]. 

In our investigation, stroke was identified in 2.5% of the cases. In comparison, however, in a study at Al Qurayyat, only 1.8% of Bell's palsy participants reported having experienced a stroke [4]. In 1.7% of cases in the current study, head and neck tumors impacted facial nerves, causing FNP. Direct anesthesia of the facial nerve can provide quick onset while the anesthetic agent is being injected, which can explain the process of facial numbness following dental surgery [3]. Peripheral FNP was not widely observed in the past 23 years following dental surgery. Nine communications were recorded from 21 cases [4].

Our findings revealed significant associations between particular risk factors and complications with facial paralysis. This was not mentioned in other studies according to our knowledge. Hypertension was linked to eye twitching, while both hypertension and diabetes were associated with speech difficulties. Immunocompromised patients also showed a relationship with eye twitching.

The limitation of the study was its small sample size. This was because of insufficient documented information in the medical records.

## Conclusions

FNP is common in the region covered by the study, and observable causes such as Bell's palsy are more likely to be involved. The ratio was higher in women than in men. We found a significant association between risk factors and complications.

We, therefore, recommend health education sessions to increase public awareness of the prevalence, causes, risk factors, and complications of the disease and provide prevention strategies to avoid complications of facial nerve damage. Further research on the association between risk factors and complications of FNP is recommended.
